# Capturing the short-lived excited singlet state in crystals of a TADF silver(i) complex

**DOI:** 10.1039/d5cc04193g

**Published:** 2025-09-19

**Authors:** Piotr Łaski, Jakub Drapała, Radosław Kamiński, Krzysztof Durka, Dariusz Szarejko, Robert Henning, Katarzyna N. Jarzembska

**Affiliations:** a Faculty of Chemistry, University of Warsaw Żwirki i Wigury 101 02-089 Warsaw Poland katarzyna.jarzembska@uw.edu.pl; b Faculty of Chemistry, Warsaw University of Technology Noakowskiego 3 00-664 Warsaw Poland; c Center for Advanced Radiation Sources, University of Chicago Chicago Illinois 60637 USA

## Abstract

Light-induced structural changes in crystals of a luminescent silver(i) complex were evaluated at 100 K *via* time-resolved laser-pump/X-ray-probe Laue diffraction. Based on theoretical modelling, they are attributed to the S_0_ → S_1_ LLCT electronic transition. Low-temperature photoluminescence spectroscopy revealed 2-ns-lived emission followed by phosphorescence. Above 200 K, the system becomes majorly TADF-emissive.

## Abstract 2

Light-induced structural changes in a TADF-exhibiting silver(i) complex were evaluated *via* time-resolved laser-pump/X-ray-probe Laue diffraction. LLCT leads to an asymmetric Ag atom shift in the short-lived S_1_ singlet excited state.

## Graphical abstract

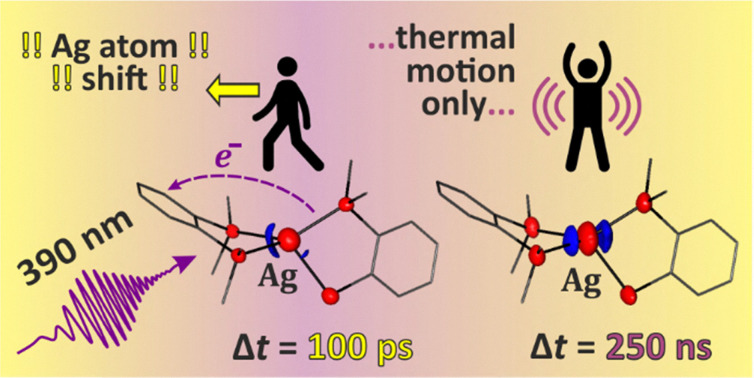

Coinage-metal (*i.e.* Au, Ag, Cu) complexes with d^10^ electronic configurations have attracted considerable attention as luminescent dopants for light-emitting diodes, being a lower cost and toxicity alternative to rare-metal coordination compounds.^1–5^ They possess full d orbitals, and thus the internal quenching of low-lying d–d* states does not occur, which makes them promising candidates for highly-emissive systems. Some of such complexes are characterized by a small singlet–triplet energy gap, so both singlet and triplet excitons can be efficiently formed under external conditions. If this is the case, a thermally activated delayed fluorescence (TADF) can be observed.^6–10^ This phenomenon has been reported to date for numerous copper(i) complexes^11,12^ but can also be exhibited by silver(i) and gold(i) coordination compounds.^13–15^

In this contribution we have examined a model silver(i) complex: [Ag(dppbz)(dpps)] (dppbz = 1,2-bis(diphenylphos-phino)benzene,^16,17^ dpps = 2-(diphenylphosphino)benzene-thiolate^18^) (hereafter AgPPPS; Fig. 1) first reported by Osawa *et al.*^19^ The compound is known to exhibit bright TADF, which arises from ligand-to-ligand charge transfer (LLCT) in the excited state and is possibly due to energetically-close-lying excited singlet and triplet states. While photophysical properties of such compounds have been widely studied, the structural dynamics in the solid state remain largely unexplored. Experimental information on structural changes and charge transfer occurring on excitation is relevant for our understanding of the phenomena which govern the properties of materials, for verification of theoretical presumptions, and for rational design of new materials. As a part of our long-term project dedicated to tracing of light-induced excited species in coinage-metal complexes in the solid state, we undertook a challenge to catch the excited state formed in crystals of AgPPPS. Since in the original paper on this compound any photo-induced structural changes were dismissed as negligible, we wanted to verify whether such changes are indeed minor, and whether they can be experimentally observed.

**Fig. 1:**
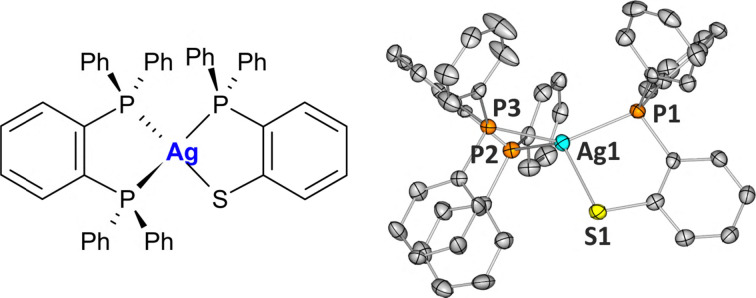
Left: Schematic drawing of the [Ag(dppbz)(dpps)] complex (AgPPPS). Right: Molecular structure of AgPPPS in the solid state. Thermal motion is shown as ellipsoids (50% probability), and labelling of carbon atoms and all hydrogen atoms are omitted for clarity.

To address this problem, we have applied the 100 ps time-resolved pump–probe X-ray Laue diffraction technique,^20–22^ time-resolved spectroscopy, and QM/MM (quantum-mechanics/molecular-mechanics) modelling of molecules in a crystal.^23^ Such combination provides a detailed view on how the molecule responds to excitation in the crystal state.

The compound features a distorted tetrahedral geometry, with the Ag^I^ core atom coordinated by two P atoms from the dppbz ligand and P and S atoms from the dpps ligand. It crystallizes in the *P*1̄ space group with one neutral molecule in the asymmetric unit (Fig. 1). The crystal structure is stabilized by numerous weak interactions, such as hydrogen-bond type interactions, including a close S1⋯H20–C20 contact (2.93 Å), several C⋯H–C edge-to-face-type interactions, as well as rather distant interactions between parallel-oriented aromatic fragments. No distinct dimeric motifs, or molecular stacking can be identified in the crystal structure.

Solid-state emission of the AgPPPS sample was previously investigated at room temperature and at 77 K by Osawa *et al.*^19^ However, in order to explicitly assess the thermal dependence of TADF effects, multi-temperature time-resolved photoluminescence measurements were conducted. Single-crystal samples were measured at five different temperatures, ranging from 100 K to room temperature, with an excitation wavelength of 390 nm. To distinguish between short- and long-lived state emissions, two different detector exposure times were used. A long exposure time of 10 ms was applied to the majority of measurements, effectively isolating the signal from long-lived states. Conversely, to capture the prompt fluorescence from short-lived states, a single measurement was conducted at 100 K using a 20 ns exposure time (Table 1). For more experimental details see the SI.

**Table 1:** AgPPPS single-crystal emission maxima (*λ*^max^_em_, also as energy values, *E*^max^_em_) and lifetimes measured by time-resolved photoluminescence spectroscopy (*T* – temperature; excitation wavelength, *λ*_ex_ = 390 nm); amplitudes (last column) are given as relative values (order is the same as lifetimes). For more details see the SI

*T*/K	*λ* ^max^_em_/nm	*E* ^max^_em_/eV	Lifetime (s), *τ*/μs	Rel. amplitudes
r.t.^a^	509	2.46	0.70(4), 3.4(1)	38%, 62%
250	509	2.46	2.08(6), 10.6(4)	60%, 40%
200	510	2.43	2.7(1), 60(2)	67%, 33%
150	521	2.38	36(1), 245(16)	62%, 38%
100	525	2.36	63(12), 482(27)	13%, 87%
100^b^	491	2.53	1.89(4) ns^c^^d^	

a Room temperature (∼296 K).

b Measurement performed within the first 20 ns to determine the fluorescence lifetime.

c Note the unit here is nanosecond.

d Mono-exponential fit.

Lowering the temperature led to an increase in the emissive state lifetimes, as the emission gradually shifted from predominantly TADF-related singlet emission to phosphorescence-dominated emission from triplet states stabilized at lower temperatures. This transition was further confirmed by the progressive red-shift of the emission spectrum at lower temperatures. Notably, a significant change in both the emission lifetimes and peak position was observed between 200 and 150 K, indicating a sharp decline in TADF efficiency within this temperature range (Table 2).

**Table 2:** Bond lengths (all in Å) around the silver atom given for different electronic states – experimental (GS – ground singlet S_0_ state, ES_100ps_ – excited state determined 100 ps after light excitation) and theoretical values (theor. data are given for the QM/MM-optimized and isolated optimized molecules) (values in square brackets); DFT(B3LYP)/6-31G**-def2-QZVPP

Bond	Experiment		Theory		
	GS (S_0_)	ES_100ps_	S_0_	S_1_	T_1_
Ag1–S1	2.535(1)	2.642(12)	2.543 [2.545]	2.728 [4.183]	2.592 [2.622]
Ag1–P1	2.447(1)	2.620(9)	2.509 [2.499]	2.557 [2.487]	2.497 [2.483]
Ag1–P2	2.5119(9)	2.348(5)	2.594 [2.564]	2.556 [2.542]	2.593 [2.563]
Ag1–P3	2.494(1)	2.369(5)	2.558 [2.592]	2.555 [2.534]	2.562 [2.597]

The short exposure time measurement at 100 K revealed a short-lived emissive state (*τ* < 2 ns), which was otherwise overshadowed by the long-lived phosphorescence emission. The decay of this short-lived emission is best described by a mono-exponential function, in contrast to the bi-exponential decay observed for the phosphorescence. This simpler decay kinetics, combined with short lifetime and small Stokes shift (101 nm), strongly suggests an emission from a singlet excited state – most likely the S_1_ state. The assignment is further supported by the solid-state absorption spectrum, which reveals that the lowest-energy peak is present at 390 nm, matching the excitation wavelength used in photoluminescence measurements (SI).

Density functional theory (DFT) calculations for the compound were performed using the B3LYP functional^24–26^ with the def2-QZVP basis set.^27,28^ Time-dependent DFT calculations were conducted for an isolated ground-state molecule. The experimentally-determined molecular geometry was used as a starting point and was optimized prior to the TDDFT calculations. The lowest electronic transitions, *i.e.* the first singlet–triplet transition at 468 nm and the first singlet–singlet at 463 nm are closely located in terms of energy and are both dominated by a pure HOMO → LUMO (highest occupied and lowest unoccupied molecular orbitals) transition.

Molecular orbital analysis revealed that the HOMO is primarily localized on the dpps ligand, while the LUMO is centred mainly on the dppbz ligand. This confirms the LLCT character of the low-energy transitions, and combined with the small energy gap, suggests the potential for TADF effects.

In the next step, we verified the actual impact that the electronic excitation has on the molecular structure of AgPPPS, since it was deemed as negligible in the original paper. Therefore, QM/MM optimization of the excited states was performed. The results indicated that the LLCT-induced electron density shift leads to a contraction of the Ag⋯dppbz-ligand distance, accompanied by an elongation of the Ag⋯dpps-ligand distance. The structural differences between the ground and excited states are best represented by the Ag1–S1 bond length, which increases by 0.185 Å for the S_0_ → S_1_ transition and by only 0.049 Å for the S_0_ → T_1_ transition. This indicates that the S_1_ state, responsible for immediate fluorescence and TADF emission, exhibits distinct structural parameters compared to the lowest-energy excited triplet state, which governs phosphorescence.

Furthermore, isolated-molecule optimizations proved insufficient for predicting excited-state structural changes. Without the crystal environment constraints simulated by the QM/MM approach, the S atom remained excessively labile, leading to dissociated structures (SI). This behaviour resembles the labile nature of the Au–S bond, noted by Osawa *et al.*^19^ In the case of AuPPPS, however, this could be attributed to the extended Au1–S1 bond length, whereas for AgPPPS, the Ag1–S1 bond length falls well within the typical range for this class of molecules (SI).^29,30^ Nevertheless, it seems that the Ag–S bond may also have the potential to undergo dissociation upon excitation, at least under vacuum.

To capture the structural differences between the S_1_ and T_1_ excited states, time-resolved laser-pump/X-ray-probe Laue diffraction experiments were conducted at the BioCARS 14-ID-B beamline of the Advanced Photon Source synchrotron (Chicago, USA).^31,32^ Single-crystal samples, mounted onto the single-axis goniometer and cooled down to 100 K, were exposed to synchronized 390 nm laser pulses (≈40 ps) and *ca.* 80 ps X-ray pulses (pink Laue beam, 15 keV at max.). Data collection involved sequential measurements (light-ON/OFF series) at two pump–probe time delays: 100 ps, primarily probing the S_1_ state, and 250 ns, expected to involve the T_1_ state. For each time delay, X-ray diffraction signals were collected both after the laser excitation (light-ON) and without it (light-OFF), with further analysis based on intensity (response) ratios: *η* = (*I*^ON^ − *I*^OFF^)/*I*^OFF^ = *R*^ON/OFF^ − 1 (SI).^20,21,33–44^ Data integration was performed using a GPU-accelerated 1D seed-skewness algorithm (SI).^45^ The five best datasets for each delay were merged, resulting in data completeness levels of about 47% and 39%, respectively. The corresponding photodifference maps, illustrating electron-density changes, are shown in Fig. 2.

**Fig. 2:**
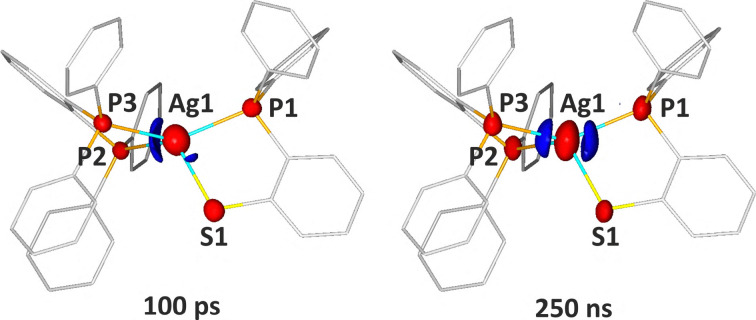
Photodifference (*F*^ON^ − *F*^OFF^) maps derived from the Laue experiment indicating electron-density changes 100 ps and 250 ns after excitation plotted on the AgPPPS ground-state geometry. Isosurfaces represent regions with electron density difference of at least ±0.35 e Å^−3^ (left) or ±0.55 e Å^−3^ (right) (blue – positive, red – negative).

In both cases, a significant negative signal indicative of electron-density distribution change appears at all heavy atom positions. This is a sign of increased atomic thermal motion and a characteristic feature of correctly processed data used for photodifference map generation (*i.e. F*^ON^ − *F*^OFF^, SI).^21,37^ The main distinction between the two datasets lies in the symmetry of the electron density influx regions. In the 100 ps data, a prominent peak is observed on the dppbz-ligand side, while the dpps-ligand side shows a weaker response. Conversely, in the 250 ns data, the electron-density changes form a nearly symmetric pair of peaks on both ligand sides of the central Ag atom.

The electron-density redistribution observed at 100 ps suggests underlying structural changes, which were evaluated for the central Ag atom based on a response-ratio structural refinement. The refined model indicates elongation of the Ag1–S1 and Ag1–P1 distances by 0.11(1) Å and 0.17(1) Å, respectively, while the Ag1–P2 and Ag1–P3 distances decrease by 0.164(6) Å and 0.125(6) Å, respectively. The excited-state population was estimated at 0.5% (SI).^46^ The photo-Wilson plot analysis^41,47,48^ suggests a temperature increase of less than 1 K upon photo-excitation.

For 250 ns delay, no distinct structural changes could be resolved, preventing reliable structural refinement. The low excited-state population and minimal temperature effects largely result from the relatively low laser power (<4 μJ per pulse) required to prevent sample degradation, as determined in preliminary tests. Consequently, in the case of experiments at longer pump–probe delays, the excited-state population could fall below the detection threshold.

Overall, our experimental findings demonstrate noticeable structural changes in the AgPPPS complex upon excitation with 390 nm laser light at 100 K. The TR X-ray Laue diffraction experiments reveal a shift of the central Ag^I^ atom towards the dppbz ligand in the excited state, with quantifiable changes in interatomic distances. This asymmetric redistribution of charge, particularly pronounced at the 100 ps time delay, provides direct (experimental) structural evidence of the LLCT process. During temperature-dependent spectroscopic measurements, by employing a 20 ns detector exposure time at 100 K, we were able to isolate and characterize this short-lived state as a singlet state, distinguishing it from the longer-lived triplet state.

The theoretical calculations attribute the observed structural changes to those of an S_1_ excited state, based on their coherence with the predicted geometry. This leads to a clear ‘chemical’ interpretation of the observed transition: the electron moves from the dpps to the dppdz ligand, which results in the shift of the (formally +1 charged) Ag^+^ centre towards the already more negatively charged dppdz ligand. Furthermore, computational investigations demonstrate that modelling of the excited-state properties requires explicit consideration of the crystal environment, *e.g.* through the QM/MM approach. Isolated-molecule calculations failed to reproduce the observed structural changes, highlighting the role of packing effects in modulating the photophysical behaviour of solid-state systems.

Overall, the study improves our understanding of photo-induced structural dynamics in coordination compounds. It demonstrates the power of TR X-ray Laue diffraction for detecting and characterizing short-lived excited states, also when very poorly populated (here the ES population is even lower – 0.5% – than in our recent paper^49^). As the field moves towards ultrafast studies with X-ray free-electron lasers, our approach and findings provide insights for future investigations into the relationships between electronic structure and molecular geometry, in TADF-active materials in particular.

P. Ł., D. S. and K. N. J. thank NSC (Poland) for funding (2020/38/E/ST4/00400, SONATA BIS). For the purpose of OA, the authors had applied a CC-BY public copyright license to any AAM version arising from this submission. XRD experiments were co-financed by EU (POIG.02.01.00-14-122/09, ERDF). WCSS is acknowledged for providing computational facilities (grant no. 285). The research used resources of APS, a U.S. DoE facility operated by ANL (DE-AC02-06CH11357). BioCARS is supported by NIH (R24GM111072). The TR setup at Sec. 14 was funded in part through collaboration with P. Anfinrud (NIH). We thank V. Stsiapura (Warsaw, Poland) for valuable help and discussions regarding spectroscopic measurements.

## Conflicts of interest

There are no conflicts to declare.

## Supplementary Material

CC-061-D5CC04193G-s001

CC-061-D5CC04193G-s002

## Data Availability

The data supporting this article have been included as part of the supplementary information (SI). Supplementary information: synthesis, XRD measurements, spectroscopic measurements, theoretical calculations, Laue data collection and processing. See DOI: https://doi.org/10.1039/d5cc04193g. In accordance with the OA policy, the high-volume raw and partially processed data are deposited in the UW Research Data Repository under the following DOI: https://doi.org/10.58132/otvuyf. CCDC 2472972 and 2472973 contain the supplementary crystallographic data for this paper.^50*a*,*b*^
