# Supplementary material for: Capturing the short-lived excited singlet state in crystals of a TADF silver(i) complex
Source: Chem Commun (Camb). 2025 Sep 19;61(85):16560–3. doi: 10.1039/d5cc04193g (PMC12489737; doi:10.1039/d5cc04193g)
Supplement: CC-061-D5CC04193G-s001 [file CC-061-D5CC04193G-s001.pdf]

Supplementary Information (SI) for Chemical Communications.
This journal is © The Royal Society of Chemistry 2025

ELECTRONIC SUPPLEMENTARY INFORMATION

Capturing the short-lived excited singlet state in crystals of a TADF silver(I) complex

Piotr Łaski,[a] Jakub Drapała,[a,b] Radosław Kamiński,[a] Krzysztof Durka,[b] Dariusz Szarejko,[a] Robert Henning,[c] Katarzyna N. Jarzembska *,[a]

[a] University of Warsaw, Faculty of Chemistry, Żwirki i Wigury 101, 02-089 Warsaw, Poland

[b] Faculty of Chemistry, Warsaw University of Technology, Noakowskiego 3, 00-664 Warsaw, Poland

[c] Center for Advanced Radiation Sources, University of Chicago, Chicago, Illinois 60637, United States

* Corresponding author: Katarzyna N. Jarzembska (katarzyna.jarzembska@uw.edu.pl)

Table of Contents

1. Synthesis **2**
2. X-ray crystallography **2**
2.1. Crystallization and diffraction 2
2.2. CSD search 3
3. Theoretical calculations and modelling **4**
4. Solid-state spectroscopy **9**
5. Time-resolved X-ray Laue diffraction **13**
5.1. Data collection 13
5.2. Data processing 13
5.3. Photodifference maps 14
5.4. Refinement 15
6. References **21**

1. Synthesis

Sodium 2-(diphenylphosphino)benzenethiolate (Na(dpps)) in Et_2O (10 mL) was prepared from sodium hydride (0.028 g, 1.2 mmol) and 2-(diphenylphosphino)-benzenethiol (dppsH) (Saxon *et al.*, 2000) (0.34 mg, 1.2 mmol). The consecutive addition of $AgCF_3SO_3$ (0.30 g, 1.2 mmol) and 1,2-bis(diphenylphosphanyl)benzene (dppbz) (Hatakeyama *et al.*, 2009, Csákÿ & Molina) (0.52 g, 1.2 mmol) afforded yellow suspension. It was filtered and the obtained solid was washed with Et_2O, and dried under vacuum to give colourless solid. Yield: 0.92 g, 93%. ^1H NMR (400 MHz, $CDCl_3$): δ = 7.70 (t, J = 7.0 Hz, 1H), 7.05–7.45 (m, 34H), 6.93 (m, 2H), 6.72 (t, J = 7.5 Hz, 1H) ppm. Anal. Calculated for $C_{48}H_{38}AgSP_3$: C, 68.01; H, 4.52; S, 3.78. Found C, 67.89; H, 4.37; S, 3.85. The NMR analyses are consistent with those reported in the literature (Osawa *et al.*, 2013).

2. X-ray crystallography

2.1. Crystallization and diffraction. Crystals suitable for X-ray diffraction were obtained via the vapour diffusion method (DMSO-solution/H_2O-diffusion). All X-ray diffraction measurements were performed on a Rigaku Oxford Diffraction SuperNova diffractometer equipped with an Atlas CCD detector, copper microfocus X-ray source (Cu-K_α radiation, λ = 1.54184 Å), multilayer optics and a low-temperature nitrogen open-gas-flow Oxford Cryosystems device (Oxford Cryostram 700 Series). Optimal data-collection strategies and further data processing (unit-cell determination, raw diffraction image integration, data scaling and absorption correction application *etc.*) were carried out using the native diffractometer *CRYSALISPRO* software suite (ver. 1.171.40 and later, 2019–2024). Structures were solved using an intrinsic phasing method as implemented in the *SHELXT* program (Sheldrick, 2015) and refined with the *JANA* package (Petříček *et al.*, 2014) within the independent atom model (IAM) approximation. Hydrogens were placed geometrically in the ideal positions and the C–H bond distances were all set to 0.96 Å. In all cases the riding model for the hydrogen thermal-motion parameters was applied ($U_{\mathrm{iso}}^{\mathrm{H}} = 1.2 \cdot U_{\mathrm{eq}}^{\mathrm{C}}$). Crystallographic data are provided free of charge by the joint Cambridge Crystallographic Data Centre (CCDC) (Allen, 2002, Groom *et al.*, 2016) and FIZ Karlsruhe – Leibniz-Institut für Informationsinfrastruktur (Zagorac *et al.*, 2019) Access Structures service. The CIF files can also be retrieved from ESI. Selected data collection, processing and refinement parameters for a single structure (CCDC code: 2472973) are shown in Table S1. Note we collected data for two crystals (CCDC for the second structure:

2472972) to explore the origin of the larger ellipsoids present at one of the phenyl ring attached to the P3 atom. It was found it is indeed possible to refine the disorder as two partially overlapping rigid bodies. However, for the sake of further analyses we used the original data (note we checked the photodifference and response-ratio refinement results – see further – for both reference monochromatic data sets and no significant differences were found).

Table S1. Selected X-ray data collection, processing and refinement parameters for the studied **AgPPPS** compound.

Moiety formula	$C_{48}H_{38}AgP_3S$	γ / °	81.388(3)
Moiety formula mass, M / a.u.	847.7	V / Å^3	1961.13(14)
Crystal system	triclinic	d_{calc} / g·cm^{-3}	1.4355
Space group	$P\bar{1}$ (No. 2)	θ range	1.79–33.09°
Z	2	Absorption coefficient, μ / mm^{-1}	0.724
F_{000}	868	No. of reflections collected / unique	46934 / 13666
Crystal colour & shape	colourless block	R_{mrg}	9.41%
Crystal size / mm^3	0.05 × 0.14 × 0.21	No. of reflections with $I > 3\sigma(I)$	5281
T / K	100	No. of parameters	478
a / Å	10.8455(5)	$R[F]$ ($I > 3\sigma(I)$)	4.77%
b / Å	12.0698(5)	$wR[F^2]$ (all data)	11.85%
c / Å	15.9133(5)	$\varrho_{res}^{min/max}$ / e·Å^{-3}	−1.11 / +2.27
α / °	72.271(3)	CCDC code	2472973
β / °	88.798(3)		

2.2. CSD search. Short summary of Cambridge Structural Database (CSD, ver. 6.00, April 2025, *CONQUEST* program, ver. 2025.1.1, build 445489) (Allen, 2002, Groom *et al.*, 2016) search performed to study Ag···S and Au···S bond lengths. In all cases 'any' bond between atoms was assumed, and only the distances were listed. Crystal structures with '3D coordinates determined', 'Only Non-disordered' and with 'No errors' were considered. For Ag···S query 1956 crystal structures were found which yielded 8486 distances. For Au···S query 1746 crystal structures were found which yielded 5259 distances. Further analysis were done within the *MERCURY* program (Macrae *et al.*, 2008).

3. Theoretical calculations and modelling

The **AgPPPS** compound related computations were carried out using the DFT method with the B3LYP functional (Becke, 1993, Lee *et al.*, 1988, Miehlich *et al.*, 1989) and a mixed basis set: 6-31G** for P, C, and H atoms, and def2-QZVPP for the Ag atom (abbreviated as 6-31G**-def2-QZVPP). These calculations, performed with the *GAUSSIAN* package (ver. 16, rev. C.01) (Frisch *et al.*, 2016), were aimed at determining the energies of potential electronic transitions, along with the corresponding molecular orbitals and geometries. Optimized molecular geometries underwent additional force constant and vibrational frequencies calculations in order to confirm their total energy successful minimum convergence. Concurrently, DFT calculations involving a simpler theory level consisting of a similar B3LYP functional and a mixed 6-31G** (P, C, H atoms) and LANL2DZ (Ag atom) basis set were also conducted (abbreviated as 6-31G**-LANL2DZ), which resulted in qualitatively accordant results. Nonetheless, for all descriptive purposes in this work, the aforementioned more sophisticated basis set was considered. For all calculations, the Grimme's dispersion correction with Becke-Johnson damping (Grimme, 2004, 2006) was also applied.

The lowest-energy electronic transitions were those occurring towards the first triplet and first singlet state, *i.e.* $S_0 \rightarrow T_1$ (λ = 468.61 nm) and $S_0 \rightarrow S_1$ (λ = 463.03 nm), both of which were attributed to a pure HOMO → LUMO transition. Another pair of energetically close transitions were the $S_0 \rightarrow T_2$ (λ = 454.20 nm) and $S_0 \rightarrow S_2$ (λ = 450.17 nm) transitions, which were attributed to a HOMO → LUMO+1 transition. It is worth noting, that the $S_0 \rightarrow S_1$ transition was characterized with an oscillator strength f = 0.0080, while the $S_0 \rightarrow S_2$ was characterized with f = 0.0023, suggesting the $S_0 \rightarrow S_1$ transition to be more than three times more likely to occur during excitation. Consecutive transitions, such as $S_0 \rightarrow T_3$ or $S_0 \rightarrow S_3$ were found to be further away energetically (below 435 nm) and thus were not considered in this study. Calculated transitions have been collectively listed in Table S2. Isolated-molecule UV-Vis spectrum predicted by these transitions is shown in Figure S1.

Molecular orbital analysis has revealed that the HOMO → LUMO transition, which is the main contributor in both $S_0 \rightarrow T_1$ and $S_0 \rightarrow S_1$ transitions, exhibits a mixed MLCT and LLCT character, where the electron density is directed away from the core silver atom and the dpps ligand towards the dppbz ligand. Similarly, in the case of the $S_0 \rightarrow S_2$ and $S_0 \rightarrow T_2$, electron density is shifted from the dpps ligand towards the dppbz ligand (LLCT

character), with simultaneous d-d type transition on the core silver atom. The relevant HOMO, LUMO and LUMO+1 molecular orbitals have been visualized in Figure S2.

Geometries of isolated molecules in the S_0, S_1 and T_1 electronic states were optimized at the DFT(B3LYP)/6-31G**-Def2QZVPP level of theory. The optimized T_1 state geometry revealed a significant lengthening of the Ag1⋯S1 distance ($\Delta_{T_1-S_0}$ = 0.077 Å) when compared to the optimized S_0 ground-state geometry. The Ag1⋯S1 distance lengthening effect was drastically overestimated in the optimized S_1 state geometry ($\Delta_{S_1-S_0}$ = 1.638 Å) – resulting in an unreliably optimized structure, most likely due to the lack of inclusion of any intermolecular interactions. This overestimation was impossible to circumvent using procedural optimization, which involved optimizing the remainder of the molecule first before proceeding to optimize solely the Ag1⋯S1 distance, or when using intermediate low-complexity theory level optimization results as a starting point. The same issue is encountered when attempting to optimize (DFT(B3LYP)/6-31G**-def2-QZVPP) an analogous gold(I) **AuPPPS** molecule, also presented by Osawa *et al.* (Osawa *et al.*, 2013), in the S_1 state. However, in the case of Au⋯S, the liability can be somewhat attributed to an abnormally large bond length of 2.571 Å (CSD search for compounds containing Au⋯S bonds revealed 5259 entries with a mean of (2.324 ± 0.114) Å). This is, however, not the case for the Ag1⋯S1 bond in silver complexes (8486 entries with a mean of (2.565 ± 0.145) Å). As such, in order to better simulate the solid-state molecular environment, a quantum-mechanics/molecular-mechanics (QM/MM) approach (Kamiński, Schmøkel, *et al.*, 2010, Vreven *et al.*, 2006) was applied. In the QM/MM approach, the central molecule was modelled at the DFT(B3LYP)/6-31G**-def2-QZVPP level of theory, while the surrounding molecular shell was approximated using the Universal Force Field (UFF) (Rappé *et al.*, 1992) with Hirshfeld charges (Hirshfeld, 1977) calculated at the same level of theory. The molecular shell, comprising a cluster of molecules within a 10 Å radius of the central species, was generated using *CLUSTERGEN* software (Kamiński *et al.*, 2013), with C–H bond lengths adjusted to neutron-normalized values of 1.089 Å (Allen *et al.*, 1987, Allen & Bruno, 2010).

Importantly, for the excited-state geometry optimization, only the central molecule was considered as excitable, reflecting the expected low excited-state population during the pump-probe experiment. A significant shift of the core silver atom towards the dppbz ligand and away from the dpps ligand was predicted in the S_1 state, reflected mostly in the increase of the Ag1⋯S1 distance ($\Delta_{S_1-S_0}$ = 0.185 Å). In the T_1 state, the shift was not

as significant, although in the similar direction, resulting in a slight increase of the Ag1···S1 distance ($\Delta_{T_1-S_0}$ = 0.049 Å). The entire molecular cluster used for calculation is presented in Figure S3. The changes in core atomic distances calculated for the molecule using the QM/MM approach are collected in Table S3.

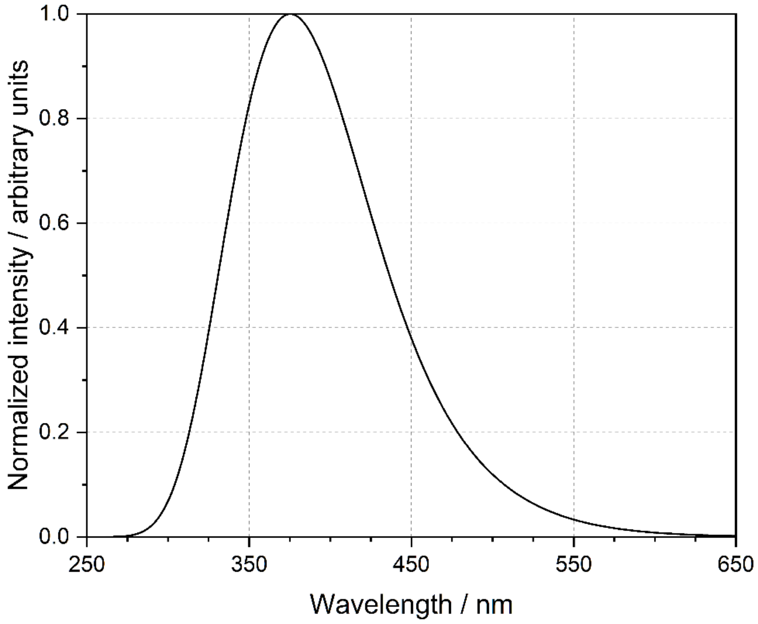

Figure S1. Normalized calculated UV-Vis spectrum computed at the DFT(B3LYP)/6-31G**-def2-QZVPP theory level for an isolated molecule.

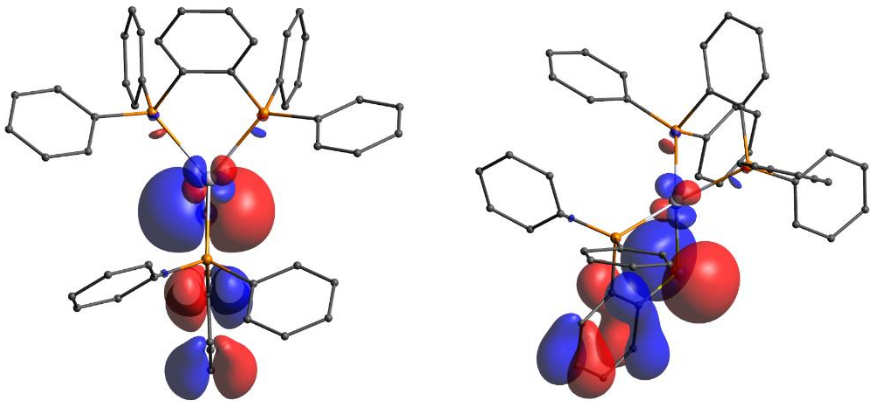

HOMO (two perspectives)

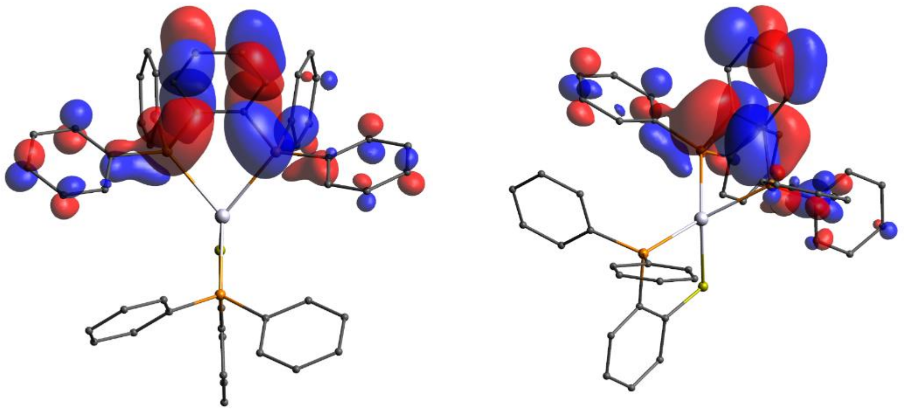

LUMO (two perspectives)

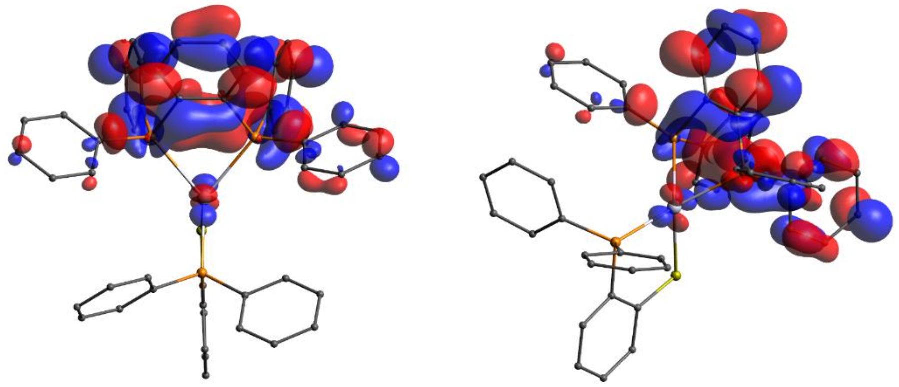

LUMO+1 (two perspectives)

Figure S2. Main molecular orbitals contributing to the $S_0 \rightarrow T_1$, $S_0 \rightarrow S_1$, $S_0 \rightarrow T_2$ and $S_0 \rightarrow S_2$ electronic transitions calculated at the DFT(B3LYP)/6-31G**-def2-QZVPP theory level, showing the MLCT and LLCT character. All orbitals are drawn at the ±0.03 $e \cdot a_0^{-3}$ contour level, blue surfaces – positive, red – negative.

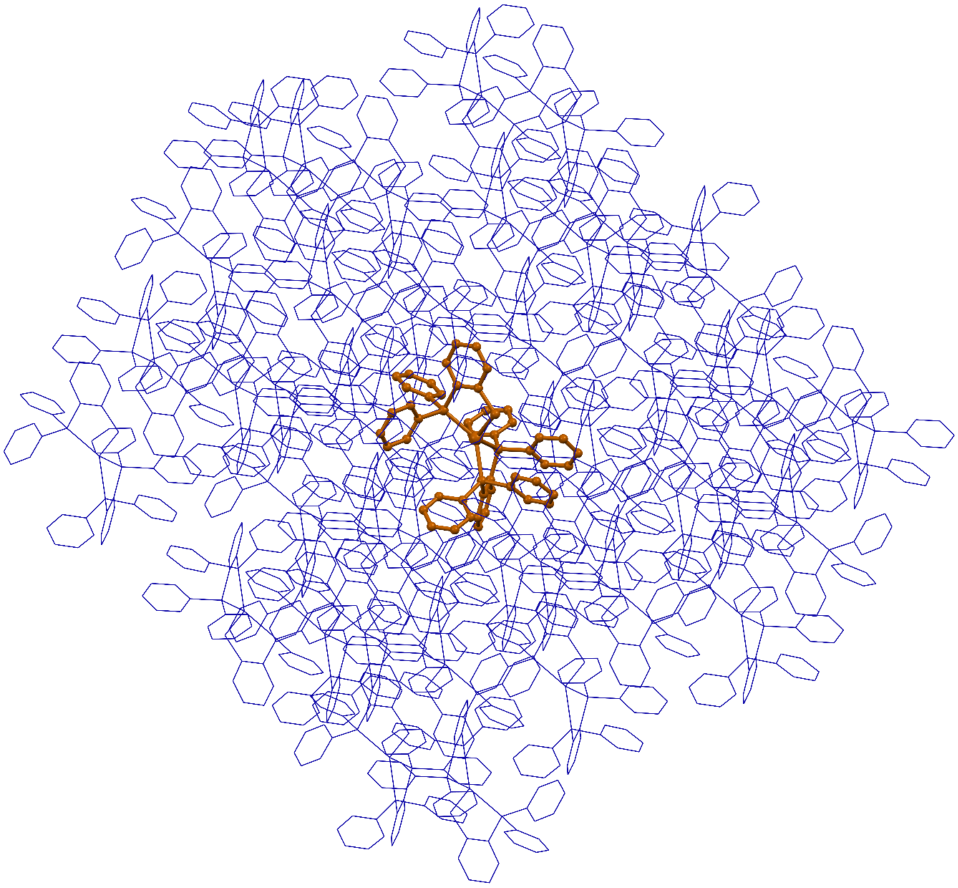

Figure S3. Molecular cluster used for QM/MM calculations. The molecule in the centre was calculated at high level of theory (DFT(B3LYP)/6-31G**-def2-QZVPP), while the molecular shell was treated at low level (UFF) level of theory. The cluster was generated so that all atoms of the central molecule have their complete atomic environment within a radius of 10 Å. The cluster involves a total of 44 molecules, which is appropriate for simulating systems with the excited-state population under 2.5%.

Table S2. Considered electronic transitions along with molecular orbitals with major molecular orbital (MO) contributions calculated at the DFT(B3LYP)/6-31G**-def2-QZVPP theory level.

Transition	*Energy, E* / eV	*Wavelength, λ* / nm	*Oscillator strength, f*	*MOs with major contributions*	*MO contribution*
$S_0 \rightarrow T_1$	2.646	468.61	0.0 [a]	HOMO → LUMO	95.79%
$S_0 \rightarrow S_1$	2.678	463.03	0.0080	HOMO → LUMO	97.93%
$S_0 \rightarrow T_2$	2.730	454.20	0.0 [a]	HOMO → LUMO+1	96.96%
$S_0 \rightarrow S_2$	2.754	450.17	0.0023	HOMO → LUMO+1	97.76%

[a] Forbidden transition.

Table S3. Change in the most important interatomic distances calculated for the QM/MM-optimized electronic states using DFT(B3LYP)/6-31G**-def2-QZVPP level of theory, compared to the observed changes in the TR Laue experiment.

Bond	*TR Laue experiment*	*QM/MM optimization*	
	$\Delta_{\mathrm{ES_{100\,ps}-GS}}$ / Å	$\Delta_{\mathrm{S_1-S_0}}$ / Å	$\Delta_{\mathrm{T_1-S_0}}$ / Å
Ag1–S1	+0.107(13)	+0.185	+0.049
Ag1–P1	+0.173(10)	+0.048	−0.012
Ag1–P2	−0.164(6)	−0.038	−0.001
Ag1–P3	−0.125(6)	−0.003	+0.004

4. Solid-state spectroscopy

The solid-state emission spectra of **AgPPPS** were measured for a single-crystal sample at various temperatures, including room temperature, 250 K, 200 K, 150 K, and 100 K, using a custom-built experimental setup at the University of Warsaw. This setup includes a tuneable Ekspla NT230 Nd:YAG DPSS laser, an Olympus IX73 inverted microscope, a Cryo Industries of America (CIA) microscope cryocooler stage, and a Princeton Instruments SP-2150 spectrograph equipped with a PI-MAX4 iCCD detector. To prepare the sample, the **AgPPPS** single crystal was carefully placed on a thin (*ca.* 0.1 mm) circular quartz plate (1 cm diameter) using a minimal amount of Type-F Olympus immersion oil. The plate was then positioned inside the microscope stage and gradually cooled to the target temperatures. The sample was excited using laser pulses at λ_{ex} = 390 nm. The resulting decay curves (Figure S4) were analysed using N-component exponential decay models, with the best fit determined based on residual analysis. The fitted function is of the following form:

$$I(t) = \sum_{i=1}^{N} \alpha_i e^{-t/\tau_i} + \beta,$$

where, in our case, N = 1 or 2 (single or double exponential decay model, respectively), α_i's are amplitudes, are τ_i's lifetimes and β is background (assumed constant). Fitted values are shown in Table S4. Table 1 in the main text shows the relative amplitude values:

$$\alpha_i^{\mathrm{rel}} = \frac{\alpha_i}{\sum_i^N \alpha_i}.$$

Stationary emission spectra at each temperature are presented in Figure S5. The maximum emission wavelength ($\lambda_{\mathrm{em}}^{\mathrm{max}}$) was estimated by averaging the peak wavelength

from the first ⅙ of the recorded frames. However, due to the irregular shape of the emission curve near its maximum and the non-uniform morphology of the crystals, this approximation has an uncertainty of approximately ±5 nm.

For the 100 K temperature point, an additional measurement was conducted, in which a 20 ns exposure-time window was used instead of the 10 ms applied in the other measurements. This allowed to differentiate the signal coming from the sample during the first 20 ns after photoexcitation, which was otherwise impossible to detect in an experiment utilizing larger exposure time. The resulting spectrum is shown in Figure S6.

Solid-state absorption spectrum was measured using the same experimental setup (laser used as a tuneable light source, Si photodiode was used as a detector) for a sufficiently thin single-crystal sample, resulting in a spectrum shown in Figure S7. The lowest-energy peak can be seen around 390 nm, which corresponds to the excitation wavelength used in the TR-Laue experiment.

Table S4. Exact numeric parameters for the converged N-component (N = 1 or 2) exponential decay functions used for modelling of luminescence decay curves at different temperatures.

T / K	α_1	τ_1 / μs	α_2	τ_2 / μs	β
r.t. [a]	0.37(2)	0.70(4)	0.62(2)	3.4(1)	0.016(2)
250	0.67(1)	2.08(6)	0.45(1)	10.6(4)	0.044(2)
200	0.65(1)	2.7(1)	0.32(1)	60(3)	0.0257(9)
150	0.59(2)	36(1)	0.35(1)	254(16)	0.051(4)
100	0.11(2)	63(12)	0.772(9)	482(27)	0.10(1)
100 [b]	1.15(4) [c,d]	1.89 ns [c,d]	–	–	0.058(1)

[a] Room temperature (≈ 296 K). [b] Measurement performed within the first 20 ns to determine the fluorescence lifetime. [c] Note the unit here is *nanosecond*. [c] Mono-exponential fit.

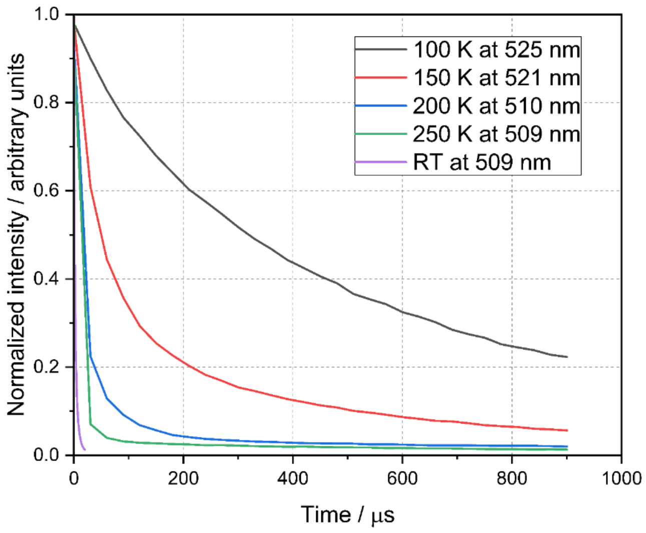

Figure S4. Luminescence maximum intensity decay curves for the **AgPPPS** single-crystal samples recorded at different temperatures. The exposure time was set to 10 ms, meaning the recorded intensity constitutes the integrated luminescence signal of the sample throughout the entire first 10 ms after photoexcitation.

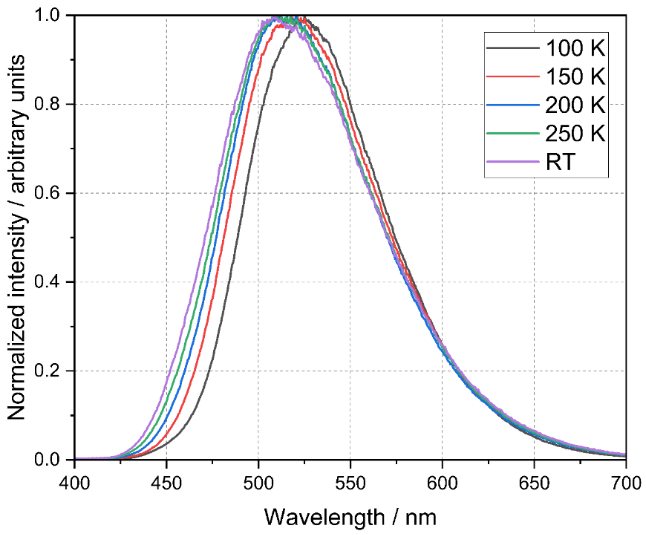

Figure S5. Emission spectra (λ_{ex} = 390 nm) for the **AgPPPS** single-crystal samples recorded at different temperatures.

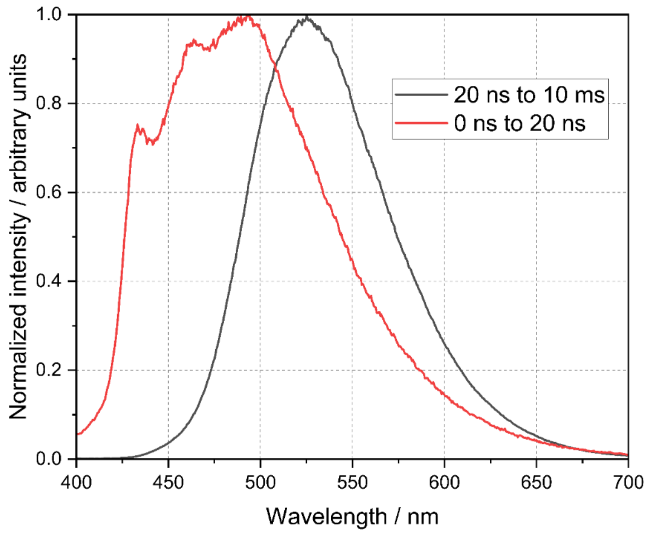

Figure S6. Stationary emission spectra (λ_{ex} = 390 nm) for the **AgPPPS** single-crystal samples recorded using an exposure time setting of 20 ns (red line) and full 10 ms (black line).

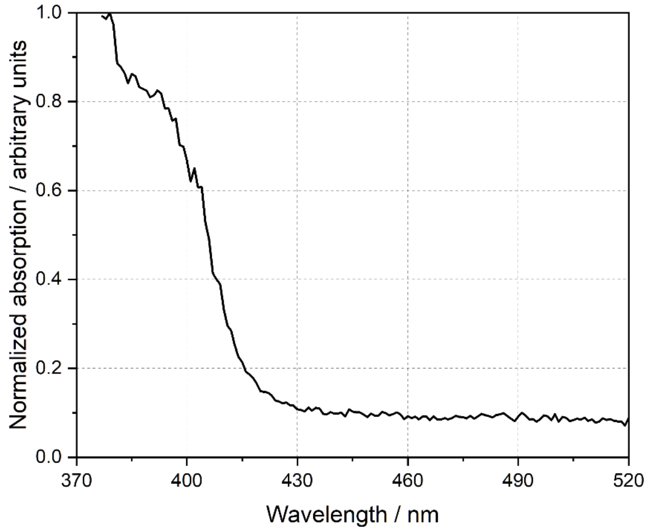

Figure S7. Absorption spectrum for the **AgPPPS** single-crystal sample. The spectrum was measured in the transmission mode at the home-made spectroscopic setup based on an inverted microscope (light source – tuneable laser, detector – Si photodiode).

5. Time-resolved X-ray Laue diffraction

5.1. Data collection. The time-resolved Laue diffraction experiments were performed using laser-pump/X-ray-probe measurements at the 14-ID-B BioCARS beamline (Graber *et al.*, 2011, Henning *et al.*, 2024) of the Advanced Photon Source (APS) in Chicago, Illinois, USA. For the measurements, the **AgPPPS** single crystals were cut to a linear size of approximately 20−50 μm, mounted on glass fibers, and maintained at a constant temperature of 100 K using a nitrogen cryostream device. The probing X-ray radiation had a polychromatic wavelength range of roughly from 0.8 to 1.0 Å (15 keV undulator setting; peak intensity of the polychromatic 'pink' beam), with each X-ray pulse lasting about 80 ps. The channel-cut scan plot, illustrating the spectral distribution of the X-ray pulses, is shown in Figure S8. Two independent time-resolved experiments have been run for **AgPPPS**, with different excitation sources and pump-probe delays.

The first experiment utilized a Ti:Sapphire picosecond laser (λ_{ex} = 390 nm, beam size (fwhm): 110 μm_h × 100 μm_v, pulse duration: 40 ps), with the laser power density ranging between 2.6 and 3.5 μJ per pulse, adjusted accordingly for each sample. The X-ray probe delay was set to 100 ps, relative to the arrival of the laser pulse on the sample. In the second experiment, an Nd:YAG nanosecond laser (λ_{ex} = 355 nm, beam size (fwhm): 80 μm_h × 105 μm_v, pulse duration: 7 ns), with the laser power density ranging between 0.3 and 2.0 μJ / pulse. In this case, the pump-probe delay was set to 250 ns.

To maximize the sample response while minimizing laser-induced deterioration, the optimal laser power setting for each sample was determined through preliminary short scans and analysis of laser-power-to-signal correlation plots (Coppens *et al.*, 2017). To compensate for long-range fluctuations in the X-ray beam's position and intensity, multiple pairs of successive light-ON and light-OFF frames were recorded at each goniometer angular setting before moving to the next position. Directly before the pump-probe experiment, each sample was also measured once in the absence of laser to facilitate further orientation-matrix determination. All of the analysed datasets were collected for different samples, as the laser radiation damage proceeded too quickly to allow multiple measurements of the same sample. The collection strategy details for all successfully acquired datasets are summarized in Table S5.

5.2. Data processing. Bragg peaks from each collected frame were localized and integrated using the *LAUEPROC* software toolkit (Szarejko *et al.*, 2020, Kamiński *et al.*, 2020), which applies a seed-skewness signal-searching algorithm optimized for time-

resolved studies of small molecules. The algorithm-specific parameters: the signal level parameter, s, and the trust level parameter, t, were semi-manually adjusted for each dataset by scanning a range of parameters and picking a pair that provides the most accurate integration masks. The parameter scan range was 0.2−2.0 for the t parameter and 0.2−3.0 for the s parameter. The internal morphological parameters were set to a 'peddh' sequence (p – isolated signal-pixel elimination, e – erosion, d – dilation, h – hole filling). After integration, reflections were indexed separately for each frame using refined orientation matrices derived from light-absent datasets, processed with a locally modified version of the *LAUEUTIL* software suite (Kalinowski *et al.*, 2012, Kalinowski *et al.*, 2011). For every pump-probe dataset, light-ON and light-OFF intensity ratios of the indexed reflections were calculated through statistical analysis of repeated (ON/OFF) pairs, employing the RATIO method (Coppens *et al.*, 2009, Coppens & Fournier, 2015a): $R_{\mathrm{o}}^{\mathrm{ON/OFF}} = I_{\mathrm{ON}}/I_{\mathrm{OFF}}$ (where 'o' denotes 'observed'). The datasets were subsequently rescaled following a published procedure (Coppens & Fournier, 2015b, Fournier *et al.*, 2016) and merged using the *SORTAV* program (Blessing, 1997, Blessing & Langs, 1987, Blessing, 1987). The statistical details of the merged datasets are presented in Tables S6, S7 & S8. Figure S9 illustrates the completeness of the collected Laue data in comparison to the full data completeness of a monochromatic experiment.

5.3. Photodifference maps. To generate the photodifference maps (Fournier & Coppens, 2014), the merged and scaled datasets were subjected to Fourier difference synthesis using $F_{\mathrm{o}}^{\mathrm{ON}}$and $F_{\mathrm{o}}^{\mathrm{OFF}}$ structure factors. The $F_{\mathrm{o}}^{\mathrm{OFF}}$values were derived from previously measured reference in-house monochromatic X-ray diffraction measurements (denoted as 'mOFF'), while $F_{\mathrm{o}}^{\mathrm{ON}}$ values (denoted from now on as $F_{\mathrm{est}}^{\mathrm{ON}}$) were estimated from the Laue experiment intensity ratios using the formula:

$$\left|F_{\mathrm{est}}^{\mathrm{ON}}\right| = \sqrt{R_{\mathrm{o}}^{\mathrm{ON/OFF}}} \cdot \left|F_{\mathrm{o}}^{\mathrm{mOFF}}\right|.$$

The photodifference maps are defined as follows:

$$\Delta\varrho_{\mathrm{pdiff}}(\mathbf{r}) = \frac{1}{V}\sum_{\mathbf{h}}\left(\left|F_{\mathrm{est}}^{\mathrm{ON}}(\mathbf{h})\right| - \left|F_{\mathrm{o}}^{\mathrm{mOFF}}(\mathbf{h})\right|\right) e^{\mathrm{i}\phi_{\mathrm{c}}^{\mathrm{mOFF}}(\mathbf{h})} e^{2\pi\mathrm{i}\mathbf{hr}},$$

where the summation includes all collected reflections, and the superscript 'c' represents 'calculated', meaning the phases are taken from the prior monochromatic experiment model. In the case of 100 ps pump-probe delay, the asymmetric nature of the positive electron density influx area around the heaviest atom suggests a structural change

occurring in the excited state. In contrast, the fully symmetric and slightly elongated positive electron density influx area present in the photodifference map of the 250 ns pump-probe delay dataset is characteristic of a lack of significant geometry changes, and can be mostly attributed to Fourier series truncation effects (*i.e.* the so-called Fourier ripples). This is most likely due to the lack of a fundamental reflection present in the collected dataset, as the 250 ns dataset, despite overall good completeness and data resolution, is missing both the 001 and 010 reflections. Additionally, a photo-Wilson plot method (Schmøkel *et al.*, 2010, Kamiński, Graber, *et al.*, 2010, Cailleau *et al.*, 2010) (Figure S10) was used to assess the temperature change upon excitation for both pump-probe delays. A temperature change upon excitation was estimated to be around 0.5 K for the 100 ps dataset, and 1.2 K for the 250 ns dataset.

5.4. Refinement. Based on the conclusions drawn from the photodifference maps, the 100 ps pump-probe delay dataset underwent a response-ratio ($\eta = R - 1$) refinement procedure using the locally-modified version of the *LASER* program (Vorontsov *et al.*, 2010). During this process, the central silver atom position, state population, and temperature scale factor were determined for the excited state measured in the Laue experiment. To enhance the signal-to-noise ratio, all experimentally obtained reflection ratios were filtered using the following criterion: $|1 - R_{\mathrm{o}}|/\sigma(R_{\mathrm{o}}) \geq 0.5$. Due to strong correlations and the assumption of a relatively low excited-state population (less than 1%), the program's automatic internal procedure failed in converging on an exact population estimate. As a result, this parameter had to be determined semi-manually by tracking variations in the program-defined R_R-factor goodness metric during refinement across different preset population values (Figure S11, Table S9). The photocrystallographic R_R-factor is defined as (Coppens *et al.*, 2010):

$$R_R = \frac{\Sigma|R_{\mathrm{o}} - R_{\mathrm{c}}|}{\Sigma R_{\mathrm{o}}},$$

where the summation includes all collected reflections. Following this method, excited-state population of the metastable state was estimated to be around 0.5%, while the temperature scale factor k_{B} (as defined here: $U_{ij}^{\mathrm{ON}} = k_{\mathrm{B}} \cdot U_{ij}^{\mathrm{OFF}}$) converged to a reasonable value of 1.05. The resulting silver-atom shift is illustrated in Figure S12.

Refinement of the 250 ns pump-probe delay dataset was attempted, although no convergence of either atomic position, excited state population or temperature scale factor could be found. This was expected, as the photodifference map already suggested

a lack of significant and systematic changes in the excited state structure, and the *LASER* program refinement procedure primarily attempts to find possible geometries which fit the collected data better than the provided ground state structure.

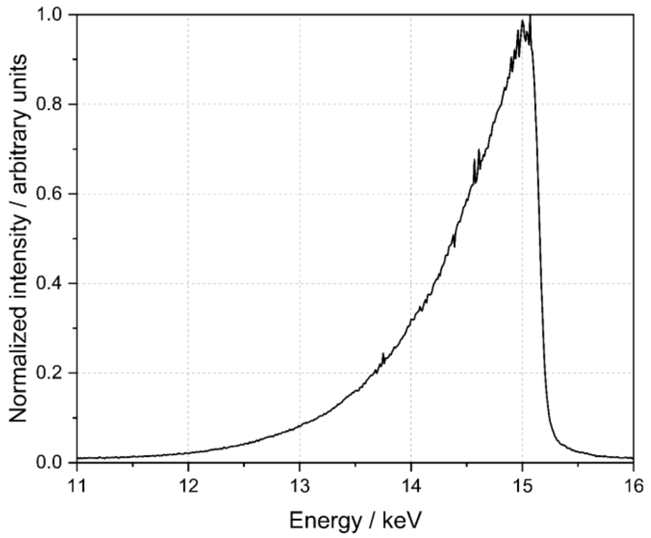

Figure S8. Channel-cut scan plot illustrating the spectral width and distribution of the 'pink' (narrow-band) X-ray beam used in the current Laue experiment.

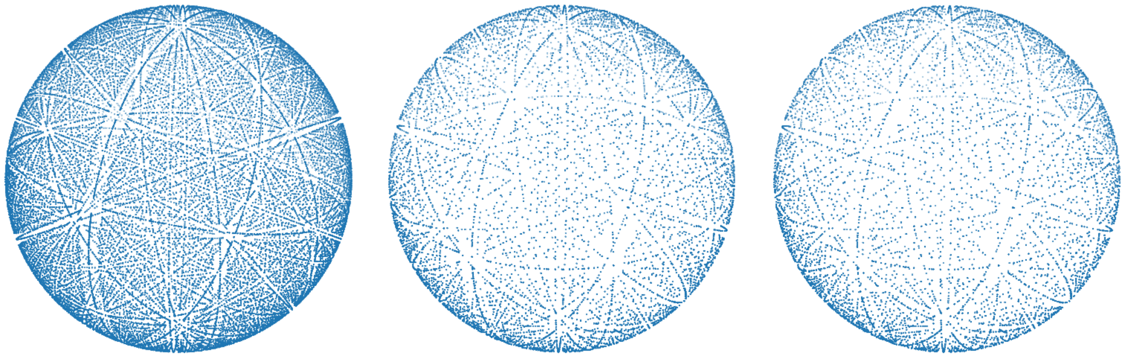

(*a*) (*b*) (*c*)

Figure S9. Visualization of all collected reflections in reciprocal space, projected onto a unit sphere. The orientation of the monochromatic sphere in (*a*) differs from that in (*b*) and (*c*), while symmetry-equivalent reflections have been generated to account for the $\bar{1}$ Laue class symmetry: (*a*) dataset collected during the in-house monochromatic experiment; (*b*) merged 100 ps delay dataset (47.2% data completeness); (*c*) merged 250 ns delay dataset (38.8% data completeness).

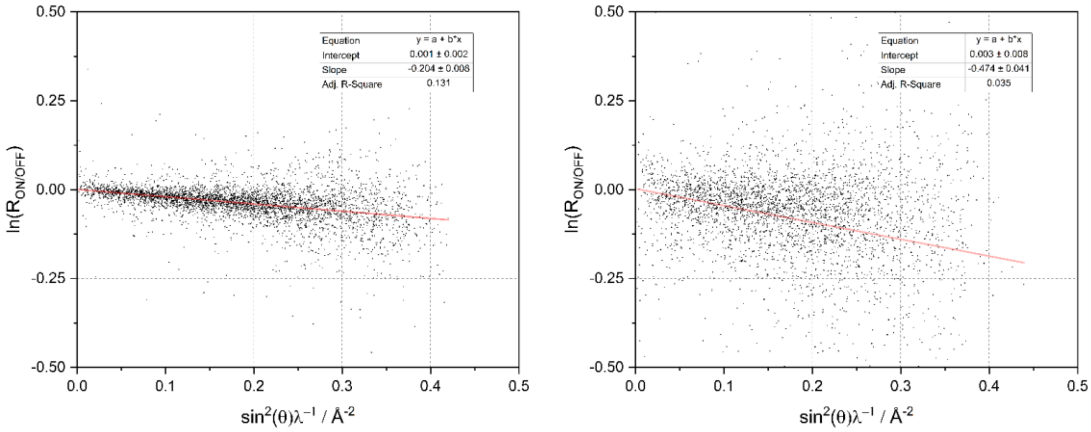

(*a*) 100 ps pump-probe delay

(*b*) 250 ns pump-probe delay

Figure S10. Photo-Wilson plots generated based on the collected TR Laue data: (*a*) the 100 ps delay dataset, where the temperature rise upon excitation can be estimated to *ca.* 0.5 K; (*b*) the 250 ns delay dataset, where the temperature rise upon excitation can be estimated to *ca.* 1.2 K.

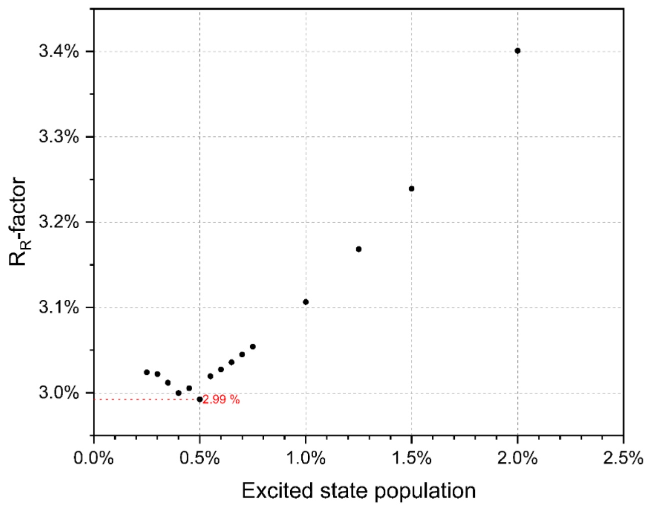

Figure S11. R_R-factor parameter obtained for each refinement model with a different set excited-state population. The lowest value (R_R = 2.99%) was obtained for the excited-state population of 0.5%.

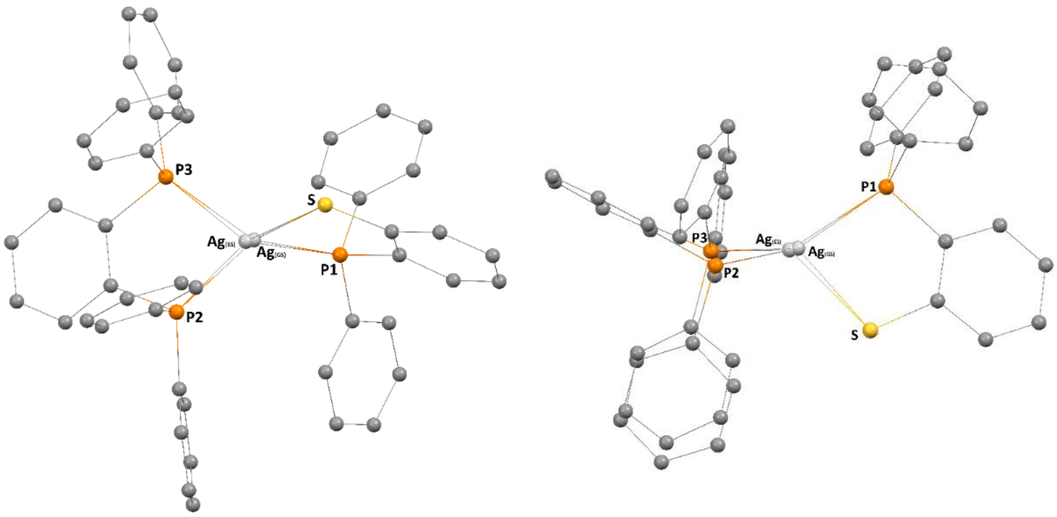

Figure S12. Refined **AgPPPS** excited-state Ag atoms' positions 100 ps after photoexcitation, plotted on the **AgPPPS** ground-state geometry, as seen from two perpendicular directions.

Table S5. Data collection strategy for the Laue experiments.

Data set No.	*Crystal size* / μm²	*Pump-probe delay*, Δt_d	*Strategy*	N_{frm}	φ_{tot} / °	φ_{inc} / °	N_{pp}	P_1 / mJ·mm⁻²	P_2 / μJ·pulse⁻¹
1	51×32	100 ps	5×(ON,OFF)	910	0–180	2.0	5	0.40	3.5
2	42×51	100 ps	5×(ON,OFF)	910	0–180	2.0	8	0.40	3.5
3	38×44	100 ps	5×(ON,OFF)	610	0–180	3.0	15	0.35	3.0
4	38×44	100 ps	5×(ON,OFF)	910	0–180	3.0	15	0.45	3.9
5	51×41	100 ps	5×(ON,OFF)	610	0–180	3.0	15	0.30	2.6
6	78×34	250 ns	5×(ON,OFF)	910	0–180	2.0	6	0.05	0.3
7	78×34	250 ns	5×(ON,OFF)	610	180–360	3.0	6	0.1	0.7
8	32×48	250 ns	5×(ON,OFF)	610	0–180	3.0	12	0.05	0.3
9	32×48	250 ns	5×(ON,OFF)	610	0–180	3.0	12	0.3	2.0
10	44×29	250 ns	5×(ON,OFF)	610	180-360	3.0	6	0.18	1.2

Table legend: N_{frm} – total number of collected frames, φ_{tot} – total angular coverage φ_{inc} – inter-frame increment, N_{pp} – number of laser-pump X-ray-probe cycles per single frame, P_1 & P_2 – laser power.

Table S6. Collected dataset statistics and maximal resolution.

Data set No.	*No. of unique reflections*	*Data completeness (in %)*	$(\sin\theta/\lambda)_{\max}$ / Å^{-1}
set 1	2627	31.0 %	0.637
set 2	2752	30.9%	0.648
set 3	2685	30.7%	0.644
set 4	2424	28.3%	0.639
set 5	2903	33.0%	0.645
merged sets 1–5	4210	47.2%	0.648
set 6	2041	23.3%	0.644
set 7	1553	19.6%	0.622
set 8	2966	32.4%	0.654
set 9	2304	24.1%	0.663
set 10	1571	17.2%	0.653
merged sets 6–10	3700	38.8%	0.663

Table S7. Distribution of measured and missing reflections in equal-volume resolution shells ($s = \sin\theta/\lambda$, $d = (2s)^{-1}$) for the 100 ps delay merged dataset.

$s_{\max}$ / Å^{-1}	$d_{\min}$ / Å	*No. of measured reflections*	*No. of missing reflections*	*Completeness* (%)
0.239	2.094	395	51	88.6
0.301	1.662	385	60	86.5
0.344	1.452	357	84	81.0
0.379	1.319	360	96	78.9
0.408	1.225	325	108	75.1
0.434	1.153	320	134	70.5
0.457	1.095	301	138	68.6
0.477	1.047	304	170	64.1
0.497	1.007	261	168	60.8
0.514	0.972	226	199	53.2
0.531	0.942	196	252	43.8
0.547	0.915	171	293	36.9
0.561	0.891	133	252	34.5
0.575	0.869	117	460	20.3
0.589	0.849	134	323	29.3
0.602	0.831	73	202	26.5
0.614	0.815	84	463	15.4
0.626	0.799	29	512	5.4
0.637	0.785	24	316	7.1
0.648	0.772	15	312	4.6

Table S8. Distribution of measured and missing reflections in equal-volume resolution shells ($s = \sin\theta/\lambda, d = (2s)^{-1}$) for the 250 ns delay merged dataset.

s_{max} / Å^{-1}	d_{min} / Å	*No. of measured reflections*	*No. of missing reflections*	*Completeness (in %)*
0.244	2.047	407	72	85
0.308	1.625	399	82	83
0.352	1.420	376	108	77.7
0.388	1.290	362	116	75.7
0.418	1.197	323	141	69.6
0.444	1.127	320	167	65.7
0.467	1.070	286	163	63.7
0.488	1.024	264	232	53.2
0.508	0.984	211	253	45.5
0.526	0.950	177	286	38.2
0.543	0.921	143	305	31.9
0.559	0.894	113	418	21.3
0.574	0.871	82	324	20.2
0.589	0.849	91	554	14.1
0.602	0.830	59	215	21.5
0.615	0.813	57	493	10.4
0.628	0.796	15	528	2.8
0.640	0.781	10	325	3
0.652	0.767	2	680	0.3
0.663	0.754	3	359	0.8

Table S9. R_R-factor calculated for each tested refinement model with differing set excited-state population 100 ps after photoexcitation. Best fit obtained for 0.5% shown in **bold red**.

ES population (in %)	*R_R- factor (in %)*
0.25	3.02
0.30	3.02
0.35	3.01
0.40	3.00
0.45	3.01
**0.50**	**2.99**
0.55	3.02
0.60	3.03
0.60	3.04
0.65	3.04
0.70	3.05
0.75	3.06
1.00	3.17
1.25	3.24
1.50	3.40

6. References

Allen, F. H. (2002). *Acta Cryst. Sect. B* **58**, 380-388.

Allen, F. H. & Bruno, I. J. (2010). *Acta Cryst. Sect. B* **66**, 380-386.

Allen, F. H., Kennard, O., Watson, D. G., Brammer, L., Orpen, A. G. & Taylor, R. (1987). *J. Chem. Soc., Perkin Trans. 2*, S1-S19.

Becke, A. D. (1993). *J. Chem. Phys.* **98**, 5648-5652.

Blessing, R. H. (1987). *Cryst. Rev.* **1**, 3-58.

Blessing, R. H. (1997). *J. Appl. Cryst.* **30**, 421-426.

Blessing, R. H. & Langs, D. A. (1987). *J. Appl. Cryst.* **20**, 427-428.

Cailleau, H., Lorenc, M., Guérin, L., Servol, M., Collet, E. & Cointe, M. B.-L. (2010). *Acta Cryst. Sect. A* **66**, 189-197.

Coppens, P. & Fournier, B. (2015a). *J. Synchrotron Rad.* **22**, 280-287.

Coppens, P. & Fournier, B. (2015b). *Struct. Dyn.* **2**, 064101.

Coppens, P., Kamiński, R. & Schmøkel, M. S. (2010). *Acta Cryst. Sect. A* **66**, 626-628.

Coppens, P., Makal, A., Fournier, B., Jarzembska, K. N., Kamiński, R., Basuroy, K. & Trzop, E. (2017). *Acta Cryst. Sect. B* **73**, 23-26.

Coppens, P., Pitak, M., Gembicky, M., Messerschmidt, M., Scheins, S., Benedict, J. B., Adachi, S.-I., Sato, T., Nozawa, S., Ichiyanagi, K., Chollet, M. & Koshihara, S.-Y. (2009). *J. Synchrotron Rad.* **16**, 226-230.

Csákÿ, A. G. & Molina, M. T. *Encyclopedia of Reagents for Organic Synthesis*.

Fournier, B. & Coppens, P. (2014). *Acta Cryst. Sect. A* **70**, 291-299.

Fournier, B., Sokolow, J. & Coppens, P. (2016). *Acta Cryst. Sect. A* **72**, 250–260.

Frisch, M. J., Trucks, G. W., Schlegel, H. B., Scuseria, G. E., Robb, M. A., Cheeseman, J. R., Scalmani, G., Barone, V., Petersson, G. A., Nakatsuji, H., Li, X., Caricato, M., Marenich, A. V., Bloino, J., Janesko, B. G., Gomperts, R., Mennucci, B., Hratchian, H. P., Ortiz, J. V., Izmaylov, A. F., Sonnenberg, J. L., Williams, Ding, F., Lipparini, F., Egidi, F., Goings, J., Peng, B., Petrone, A., Henderson, T., Ranasinghe, D., Zakrzewski, V. G., Gao, J., Rega, N., Zheng, G., Liang, W., Hada, M., Ehara, M., Toyota, K., Fukuda, R., Hasegawa, J., Ishida, M., Nakajima, T., Honda, Y., Kitao, O., Nakai, H., Vreven, T., Throssell, K., Montgomery Jr., J. A., Peralta, J. E., Ogliaro, F., Bearpark, M. J., Heyd, J. J., Brothers, E. N., Kudin, K. N., Staroverov, V. N., Keith, T. A., Kobayashi, R., Normand, J., Raghavachari, K., Rendell, A. P., Burant, J. C., Iyengar, S. S., Tomasi, J., Cossi, M., Millam, J. M., Klene, M., Adamo, C., Cammi, R., Ochterski, J. W., Martin, R. L., Morokuma, K., Farkas, O., Foresman, J. B. & Fox, D. J. (2016). *GAUSSIAN 16.*

Graber, T., Anderson, S., Brewer, H., Chen, Y.-S., Cho, H., Dashdorj, N., Henning, R. W., Kosheleva, I., Macha, G., Meron, M., Pahl, R., Ren, Z., Ruan, S., Schotte, F., Šrajer, V., Viccaro, P. J., Westferro, F., Anfinrud, P. & Moffat, K. (2011). *J. Synchrotron Rad.* **18**, 658-670.

Grimme, S. (2004). *J. Comput. Chem.* **25**, 1463-1473.

Grimme, S. (2006). *J. Comput. Chem.* **27**, 1787-1799.

Groom, C. R., Bruno, I. J., Lightfoot, M. P. & Ward, S. C. (2016). *Acta Cryst. Sect. B* **72**, 171-179.

Hatakeyama, T., Kondo, Y., Fujiwara, Y.-i., Takaya, H., Ito, S., Nakamura, E. & Nakamura, M. (2009). *Chem. Commun.*, 1216-1218.

Henning, R. W., Kosheleva, I., Šrajer, V., Kim, I.-S., Zoellner, E. & Ranganathan, R. (2024). *Struct. Dyn.* **11**, 014301.

Hirshfeld, F. L. (1977). *Theor. Chim. Acta* **44**, 129-138.

Kalinowski, J. A., Fournier, B., Makal, A. & Coppens, P. (2012). *J. Synchrotron Rad.* **19**, 637-646.

Kalinowski, J. A., Makal, A. & Coppens, P. (2011). *J. Appl. Cryst.* **44**, 1182-1189.

Kamiński, R., Graber, T., Benedict, J. B., Henning, R., Chen, Y.-S., Scheins, S., Messerschmidt, M. & Coppens, P. (2010). *J. Synchrotron Rad.* **17**, 479-485.

Kamiński, R., Jarzembska, K. N. & Domagała, S. (2013). *J. Appl. Cryst.* **46**, 540-534.

Kamiński, R., Schmøkel, M. S. & Coppens, P. (2010). *J. Phys. Chem. Lett.* **1**, 2349-2353.

Kamiński, R., Szarejko, D., Pedersen, M. N., Hatcher, L. E., Łaski, P., Raithby, P. R., Wulff, M. & Jarzembska, K. N. (2020). *J. Appl. Cryst.* **53**, 1370-1375.

Lee, C., Yang, W. & Parr, R. G. (1988). *Phys. Rev. B* **37**, 785-789.

Macrae, C. F., Bruno, I. J., Chisholm, J. A., Edgington, P. R., McCabe, P., Pidcock, E., Rodriguez-Monge, L., Taylor, R., Streek, J. v. d. & Wood, P. A. (2008). *J. Appl. Cryst.* **41**, 466-470.

Miehlich, B., Savin, A., Stoll, H. & Preuss, H. (1989). *Chem. Phys. Lett.* **157**, 200-206.

Osawa, M., Kawata, I., Ishii, R., Igawa, S., Hashimoto, M. & Hoshino, M. (2013). *J. Mater. Chem. C* **1**, 4375-4383.

Petříček, V., Dušek, M. & Palatinus, L. (2014). *Z. Kristallogr.* **229**, 345-352.

Rappé, A. K., Casewit, C. J., Colwell, K. S., III, W. A. G. & Skiff, W. M. (1992). *J. Am. Chem. Soc.* **114**, 10024-10035.

Saxon, E., Armstrong, J. I. & Bertozzi, C. R. (2000). *Org. Lett.* **2**, 2141-2143.

Schmøkel, M. S., Kamiński, R., Benedict, J. B. & Coppens, P. (2010). *Acta Cryst. Sect. A* **66**, 632-636.

Sheldrick, G. M. (2015). *Acta Cryst. Sect. A* **71**, 3-8.

Szarejko, D., Kamiński, R., Łaski, P. & Jarzembska, K. N. (2020). *J. Synchrotron Rad.* **27**, 405-413.

Vorontsov, I., Pillet, S., Kamiński, R., Schmøkel, M. S. & Coppens, P. (2010). *J. Appl. Cryst.* **43**, 1129-1130.

Vreven, T., Byun, K. S., Komáromi, I., Dapprich, S., Montgomery, J. A., Morokuma, K. & Frisch, M. J. (2006). *J. Chem. Theory Comput.* **2**, 815-826.

Zagorac, D., Muller, H., Ruehl, S., Zagorac, J. & Rehme, S. (2019). *J. Appl. Cryst.* **52**, 918-925.
